# Hsa_circRNA_0054633 is highly expressed in gestational diabetes mellitus and closely related to glycosylation index

**DOI:** 10.1186/s13148-019-0610-8

**Published:** 2019-02-08

**Authors:** Hangyu Wu, Siyang Wu, Yingchao Zhu, Mei Ye, Jun Shen, Yan Liu, Yisheng Zhang, Shizhong Bu

**Affiliations:** 1 Diabetes Research Center, Medical School, Ningbo University, 818 Fenghua Road, Ningbo, 315211 Zhejiang China; 2 Department of Gynaecology and Obstetrics, Ningbo University Medical Center Lihuili Eastern Hospital, Taipei Medical University, 1111 Jiangnan Road, Ningbo, 315048 Zhejiang China

**Keywords:** Circular RNA, Hsa_circRNA_0054633, Gestational diabetes mellitus (GDM), Biomarker

## Abstract

**Background:**

Circular RNA (circRNA) is involved in the pathological processes of various diseases. CircRNA is more stable than linear RNAs and is expressed in high levels in tissues, making it a better biomarker candidate than linear RNAs. In this study, we aimed to identify potential circRNA biomarkers of gestational diabetes mellitus (GDM).

**Methods:**

A retrospective case–control study was conducted using data and samples from women treated at a hospital in China between July 10, 2017, and February 15, 2018. We collected serum samples from 40 healthy pregnant women (controls) and 40 women with GDM (cases) during the second trimester as well as 65 controls and 65 cases during the third trimester of pregnancy. Placenta tissues and neonatal cord blood were each from another 20 cases and 20 controls. We selected six circRNAs (hsa_circRNA_0054633, hsa_circRNA_103410, hsa_circRNA_063981, hsa_circRNA_102682, hsa_circRNA_0018508, and hsa_circRNA_406918) as candidate biomarkers and used quantitative reverse transcriptase polymerase chain reaction (qRT-PCR) to measure their concentrations in the serum and placental tissues. The Pearson correlation test was used to assess the correlation between various circRNAs and between circRNA and clinical variables. The area under the receiver operating characteristic (ROC) curve was used to assess the diagnostic value of circRNAs for GDM at each stage.

**Results:**

Hsa_circRNA_0054633 was highly expressed in the blood during the second and third trimesters; its expression was also high in the placenta but low in the cord blood (*P* < 0.05). Hsa_cirRNA_0054633 was highly correlated with GHBA1 and GHBA1c levels in maternal blood samples at various stages of the GDM group (including placental tissue and umbilical cord blood) (*P* < 0.05). Hsa_circRNA_063981, hsa_circRNA_102682, and hsa_circRNA_103410 were also differentially expressed between the case and control groups at different stages (*P* < 0.05). There was a strong correlation between hsa_circRNA_0054633 and hsa_circRNA_103410 levels in third-trimester maternal blood (*P* = 0.000, *r* = 0.554) and in neonatal umbilical cord blood (*P* = 0.000, *r* = 0.866). Hsa_circRNA_0054633 showed a significant diagnostic value in the second and third trimesters of pregnancy, placenta, and cord blood (AUC = 0.793, 0.664, 0.747, and 0.783, respectively, *P* < 0.001).

**Conclusion:**

This study suggests that hsa_cirRNA_0054633 is abnormally expressed in GDM patients and may play a potential role in the development of GDM. The possibility of using circRNAs for the diagnosis of GDM requires additional investigation in future studies.

**Electronic supplementary material:**

The online version of this article (10.1186/s13148-019-0610-8) contains supplementary material, which is available to authorized users.

## Introduction

Gestational diabetes mellitus (GDM) refers to the condition of having normal glucose metabolism before pregnancy but impaired glucose tolerance and elevated fasting glucose concentrations during pregnancy [1]. The prevalence of GDM varies from country to country and even from region to region [2]. The latest epidemiological studies show that the prevalence of GDM in the USA is 9% [2], while the incidence of GDM in Asian countries is 3.0–21.2% [3]. With the increase in the rate of obesity and the increase in maternal age, the incidence of gestational diabetes is increasing [4]. Women who develop hyperglycemia during pregnancy often have increased risks of adverse pregnancy outcomes. In 2013, the International Diabetes Federation (IDF) reported that an estimated 21.4 million live births were from women with GDM [5], and these newborns were prone to macrosomia, shoulder dystocia, and neonatal hypoglycemia [5, 6]. At present, diagnosis of GDM relies primarily on the diagnostic criteria given by the American Diabetes Association (ADA) and the International Association of Diabetes and Pregnancy Study Group (IADPSG) [7]. A standard test is performed during 24 to 28 weeks of gestation, sometimes delayed to 32 weeks. Testing at this stage can often delay the diagnosis of GDM, causing serious adverse consequences for the mother and child. In addition, some biomarker detection methods, such as mid-pregnancy plasma protein profile analysis, hair metabolomics (concentration of adipic acid), and capillary blood glucose, have low diagnostic sensitivity to GDM [8–10]. Therefore, an early stage, highly sensitive biomarker of GDM is needed. Some researchers have sought to find new indicators of GDM through associations between genetic variants and GDM [11, 12], but delays the diagnosis of the disease, causing adverse consequences.

Circular RNA (circRNA) is a recently discovered endogenous non-coding RNA. Studies have found that circRNAs play important roles in the regulation of gene expression, which suggests that circRNAs have great potential in clinical diagnosis and treatment. In a circRNA, the 3′ and 5′ ends are linked to form a complete circular structure, so circRNAs are resistant to the degradation by RNA exonucleases and therefore have higher biological stability than linear RNAs. It is widely found in a variety of organisms, and a large number of circRNAs are found in humans, mice, nematodes, and plants [13]. CircRNAs play important roles in various diseases including atherosclerosis, diabetes, and cancer [14–17]. The high biological stability of circRNA is ideal for its use as a biomarker for disease. It has been reported that circRNA is involved in the regulation of insulin secretion and pathogenesis of diabetes. CircRNA-CDR1 is a natural antisense transcript of CDR1 and has been found to regulate insulin secretion and β cell renewal [18]. Yan et al. found that circRNAs were abnormally expressed in placental tissues of GDM patients and that 227 circRNAs were significantly upregulated and 255 circRNAs were significantly downregulated [16]. Although some reports have suggested that circRNAs may be involved in GDM, whether these circRNAs are potential biomarkers is not clear. Diseases often alter the mother’s physiological condition, so placental epigenetics such as circRNA expression levels may be affected by the adverse environment of GDM patients and may reflect their diet and metabolic status [19]. In addition, the origin of most GDM-related adverse pregnancy outcomes can be traced back to the placenta. Studies have found that GDM exhibits immature placental villi or changes in villus branch morphology, which ultimately leads to changes in placental morphology and function, resulting in limited intrauterine growth and increased risk of preterm birth [20]. Additionally, epigenetic changes in the peripheral blood of GDM patients might be potential biomarkers for GDM diagnosis and disease detection.

Therefore, we studied the peripheral blood of pregnant women during the second and third trimesters as well as the placenta and umbilical cord blood after childbirth. Real-time quantitative PCR was used to determine the levels of six circRNAs in the serum and placental tissues to lay a foundation for the discovery of potential early biomarkers for GDM.

## Results

### Participant characteristics

Table 1 shows the characteristics of the women and newborns in the second and third trimesters. There were no significant differences in the age, educational level, pre-pregnancy BMI, gestational age, or delivery methods between cases and controls (*P* > 0.05 for all). Table 1 also shows that there was no significant difference in the birth weight of the baby, neonatal apgar score, or the incidence of fetal distress between the control group and the GDM group (*P* > 0.05). However, the BMI of women with GDM was significantly higher than that of the controls (*P* < 0.05, Table 1). In addition, the incidence of neonatal hypoglycemia in the GDM group was significantly higher than that in the control group (*P* < 0.05).

**Table 1:** The clinical characteristics of the study population (second and third trimester mothers and neonates of the third trimester mothers)

Characteristic	Healthy control group (*n* = 40)	Gestational diabetes mellitus group (*n* = 40)	*P* value^a^
Second trimester			
Age, year	29.88 ± 3.77	31.80 ± 3.99s	0.427
High school education or lower, % (*n*)	28 (70)	26 (65)	0.633
Gestational age, weeks	24.20 ± 2.34	23.97 ± 2.45	0.900
Height, cm	160.58 ± 3.89	160.92 ± 5.40	0.792
Mid-pregnancy weight, kg	59.92 ± 11.69	64.96 ± 7.69	0.156
History of abortion, % (*n*)	13 (33)	14 (35)	0.813
Pre-pregnancy BMI, kg/m^2^	22.14 ± 2.26	23.30 ± 2.51	0.075
Mid-pregnancy BMI, kg/m^2^	23.13 ± 3.18	25.14 ± 2.50	0.014*
Third trimester	Healthy control group (*n* = 65)	Gestational diabetes mellitus group (*n* = 65)	*P* value^a^
Age, year	29.06 ± 3.66	30.04 ± 3.67	0.244
High school education or lower, % (n)	40 (62)	47 (72)	0.192
Gestational age, weeks	38.54 ± 1.98	38.09 ± 2.68	0.276
Height, cm	160.63 ± 4.84	160.09 ± 4.72	0.255
Late-pregnancy weight, kg	67.37 ± 8.27	69.47 ± 7.73	0.141
Pre-pregnancy BMI, kg/m^2^	21.25 ± 3.10	21.50 ± 2.54	0.274
Late-pregnancy BMI, kg/m^2^	26.08 ± 2.84	27.22 ± 2.74	0.022*
Gravidity	2.22 ± 1.45	2.31 ± 1.52	0.869
Parity	0.48 ± 0.59	0.40 ± 0.52	0.512
Mode of delivery, % (*n*)			
Cesarean	26 (40)	30 (46)	0.479
Vaginal	39 (60)	35 (54)	
History of abortion % (*n*)	8 (12)	9 (14)	0.795
Neonates			
Neonatal birth weight, kg	3.31 ± 0.44	3.38 ± 0.41	0.301
Apgar score			
1 min	8.95 ± 0.29	8.82 ± 0.72	0.329
10 min	9.98 ± 0.13	9.95 ± 0.22	0.311
Macrosomia and premature infants, % (*n*)	5 (7.7)	8 (12.3)	0.380
Neonatal hypoglycemia, % (*n*)	1 (1.5)	8 (12.3)	0.016*
Fetal distress, % (*n*)	1 (1.5)	4 (6.2)	0.171

*Abbreviations*: *BMI* body mass index (calculated as weight in kilograms divided by the square of height in meters)

Values are given as mean ± SD or number (percentage), unless otherwise indicated

*p value from *t* test or chi-squared test comparing means or proportions between GDM cases and controls in the current study. *P* < 0.05 was considered statistically significant

### Clinical chemical analyses

We tested a comprehensive metabolic panel, anemia index, and glycosylated hemoglobin level in the maternal serum samples (Table 2). Glycated hemoglobin levels (GHBA and GHBA1c) were higher in the GDM group than in the control group (*P* < 0.05) in both cohorts. Total cholesterol concentrations in the controls were significantly lower than those in GDM patients (*P* < 0.05). In the third-trimester cohort, the serum concentration of HDL cholesterol in the controls was significantly higher than that in the GDM group, while the APOB concentration was significantly lower than that in GDM patients (*P* < 0.05). In addition, there was no significant difference in hemoglobin, electrolyte level, anemia index, ALT, or AST between the cases and controls (*P* > 0.05).

**Table 2:** Variables associated with gestational diabetes mellitus

Variable	Healthy control group (*n* = 40)	Gestational diabetes mellitus group (*n* = 40)	*P* value^a^
Mid-pregnancy characteristics (T1)			
OGTT			
Fasting glucose, mmol/L	4.01 ± 0.31	4.81 ± 0.58	0.000*
1-h glucose, mmol/L	6.82 ± 1.27	9.83 ± 1.37	0.000*
2-h glucose, mmol/L	5.95 ± 0.95	8.54 ± 0.85	0.000*
Comprehensive metabolic panel			
ALT, U/L	16.85 ± 7.54	14.96 ± 12.30	0.064
AST, U/L	20.07 ± 4.56	18.16 ± 7.62	0.083
TBA, umol/l	2.39 ± 1.31	2.90 ± 2.01	0.306
Hemoglobin, g/L	111.69 ± 14.97	115.10 ± 9.60	0.306
Triglycerides, mmol/L	3.07 ± 1.14	3.66 ± 1.61	0.029*
Total cholesterol, mmol/L	5.70 ± 0.83	5.72 ± 0.90	0.798
HDL-C, mmol/L	1.91 ± 0.22	1.83 ± 0.32	0.721
LDL-C, mmol/L	3.43 ± 0.48	3.23 ± 0.63	0.721
GHBA1, %	6.41 ± 0.44	7.10 ± 0.90	0.003*
GHBA1c, %	4.71 ± 0.24	5.23 ± 0.56	0.001*
Three tests for anemia			
Ferritin, umol/l	21.63 ± 26.56	21.82 ± 14.73	0.55
Folic acid, nmol/L	26.57 ± 12.36	29.20 ± 16.32	0.684
Vitamin B12, pmol/L	265.39 ± 107.12	245.82 ± 55.51	0.987
Variable	Healthy control group (*n* = 65)	Gestational diabetes mellitus group (*n* = 65)	*P* value^a^
Late-pregnancy features (T2)			
GHBA1, %	–	7.25 ± 0.99	–
GHBA1c, %	–	5.45 ± 0.61	–
Hemoglobin, g/L	125.83 ± 10.8	125.09 ± 13.68	0.733
Comprehensive metabolic panel			
ALT, U/L	13.65 ± 9.23	13.69 ± 5.95	0.479
AST, U/L	20.29 ± 5.64	20.37 ± 5.87	0.887
Glucose, mmol/l	4.19 ± 0.61	4.63 ± 1.14	0.007*
Triglycerides, mmol/L	2.76 ± 0.98	3.61 ± 1.59	0.000*
Total cholesterol, mmol/L	5.80 ± 1.17	5.74 ± 1.09	0.793
HDL-C, mmol/L	1.95 ± 0.41	1.78 ± 0.34	0.009*
LDL-C, mmol/L	3.35 ± 0.84	3.28 ± 0.87	0.596
APOA1, g/L	2.26 ± 0.39	2.16 ± 0.29	0.188
APOE, g/L	1.26 ± 0.29	1.26 ± 0.28	0.993
APOB, mg/dL	8.31 ± 1.46	9.07 ± 2.03	0.018*
TBA, umol/l	3.37 ± 2.46	3.58 ± 2.37	0.241
K, mmol/l	3.93 ± 0.29	3.94 ± 0.28	0.576
NA, mmol/l	138.68 ± 1.51	138.48 ± 1.59	0.464
CA, mmol/l	2.18 ± 0.11	2.20 ± 0.11	0.686
P, mmol/l	1.22 ± 0.21	1.23 ± 0.15	0.422
Mg, mmol/l	0.71 ± 0.09	0.71 ± 0.14	0.880
Three tests for anemia			
Ferritin, umol/l	28.48 ± 18.91	28.19 ± 21.09	0.108
Folic acid, nmol/L	28.22 ± 11.59	31.52 ± 11.64	0.503
Vitamin B12, pmol/L	232.05 ± 92.71	242.25 ± 80.16	0.733

Values are given as mean ± standard deviation, unless otherwise indicated

*Abbreviations*: *OGTT* oral glucose tolerance test, *ALT* glutamic pyruvic transaminase, *AST* glutamic oxaloacetic transaminase, *TBA* total bile acid, *HDL-C* high-density lipoprotein cholesterol, *LDL-C* low-density lipoprotein cholesterol, *APOA1* apolipoprotein A1, *APOE* apolipoprotein E, *APOB* apolipoprotein B

^a^All variables were compared using an independent samples *t* test or nonparametric test; "*" stands for *P*<0.05, *P* < 0.05 was considered statistically significant

### Hsa_circRNA_0054633 was highly expressed during the second trimester and was associated with changes in glycated hemoglobin

**Fig. 1:**
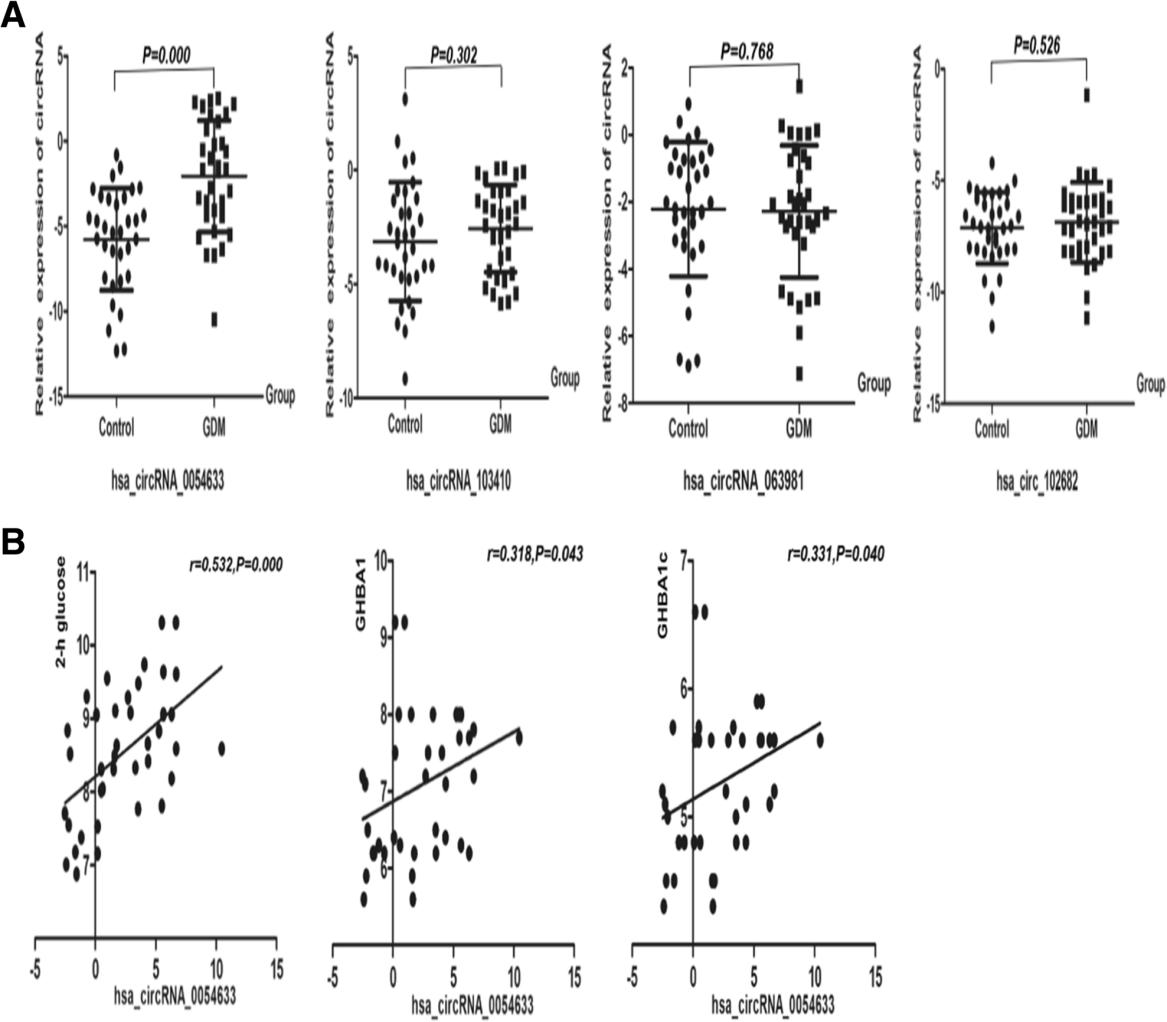
**a** Serum levels of hsa_circRNA_0054633, hsa_circRNA_103410, hsa_circRNA_063981, and hsa_circRNA_102682 in healthy pregnant women (control; *n* = 40) and patients with gestational diabetes (GDM; *n* = 40) during the second trimester. **b** Correlation analysis of the serum hsa_circRNA_0054633 level with GHBA1, GAHA1c, and 2-h glucose in the GDM group

To test the six candidate circRNAs, qRT-PCR was performed for the second-trimester cohort (Fig. 1). The level of hsa_circRNA_0054633 in GDM patients was significantly higher than in the controls (*P* < 0.001, Additional file 1: Table S2). However, there were no significant differences in the levels of other circRNAs between the two groups. Next, we analyzed the correlation between the hsa_circRNA_0054633 level and blood biochemical markers in GDM maternal serum. The level of hsa_circRNA_0054633 was significantly correlated with 2-h glucose, GHBA1, and GHBA1c, and the correlation coefficients were 0.532, 0.318, and 0.331, respectively (*P* < 0.05, Additional file 1: Table S3). Hsa_circRNA_0018508 was expressed during the second trimester, and its level in the serum was correlated with that of hsa_circRNA_0054633 (*P* < 0.05), but it was not expressed in the third trimester (Additional file 1: Figure S1). The specific cause of this is unknown.

### Hsa_circRNA_0054633 was also highly expressed in the third trimester in the GDM group and was correlated with hsa_circRNA_103410

**Fig. 2:**
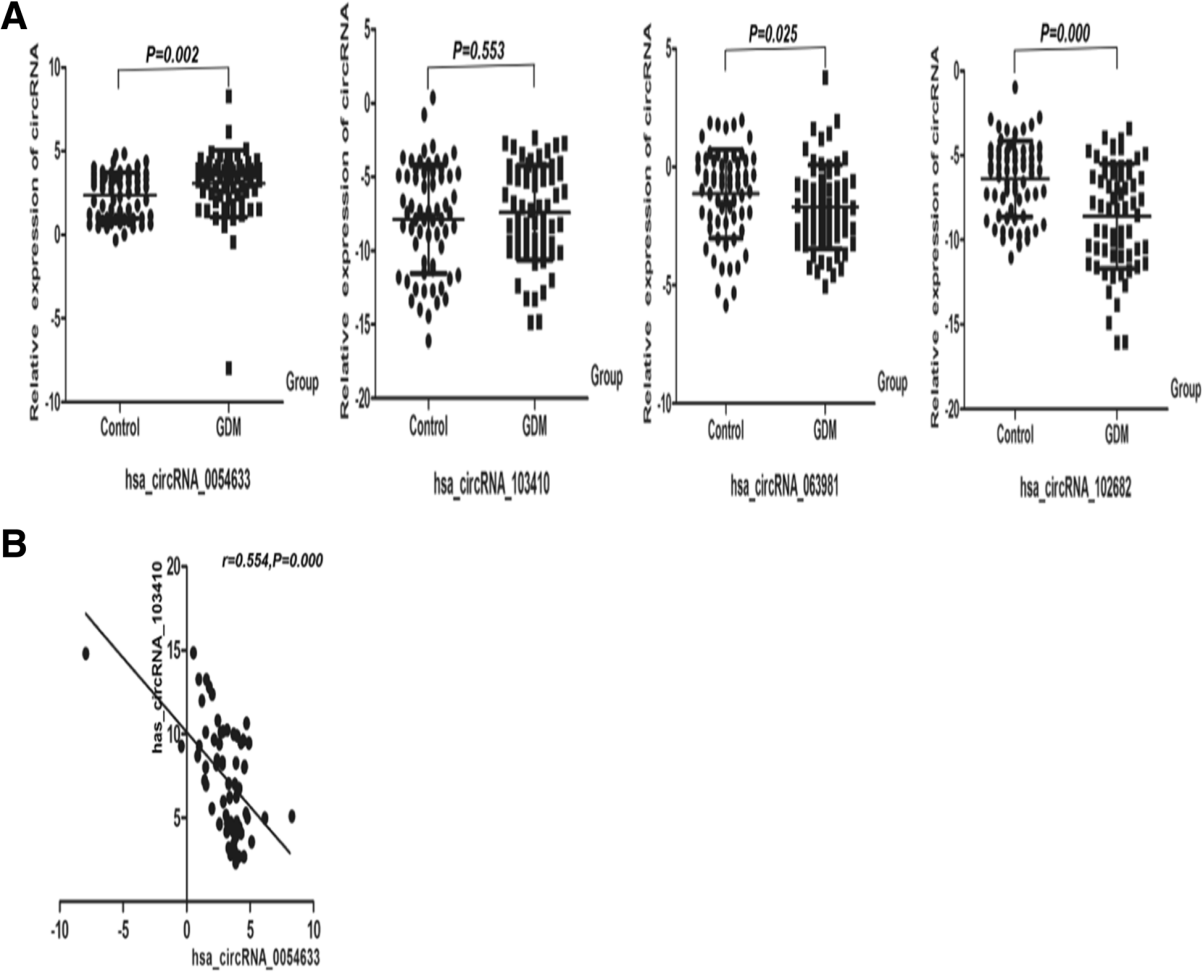
**a** Levels of circRNAs in the serum of healthy pregnant women (control; *n* = 65) and patients with gestational diabetes (GDM; *n* = 65) during the third trimester. **b** Correlation between the levels of the circRNAs in the women with GDM

By qRT-PCR, we also found that the level of hsa_circRNA_0054633 in GDM patients was significantly higher than that in the controls in the third-trimester cohort (*P* = 0.002, Fig. 2). There was a significant correlation between the level of hsa_circRNA_0054633 and the level of hsa_circRNA_103410 in the serum of GDM patients (*P* = 0.000, *r* = 0.554). However, the level of hsa_circRNA_103410 was not significantly different between the cases and controls during the third trimester of pregnancy. In addition, the levels of hsa_circRNA_063981 and hsa_circRNA_102682 in healthy subjects were significantly higher than those in the GDM group (*P* < 0.05).

### Hsa_circRNA_0054633 level was low in the umbilical cord blood of newborns in the GDM study group and was correlated with serum hsa_circRNA_103410 level and maternal glycosylated hemoglobin

**Fig. 3:**
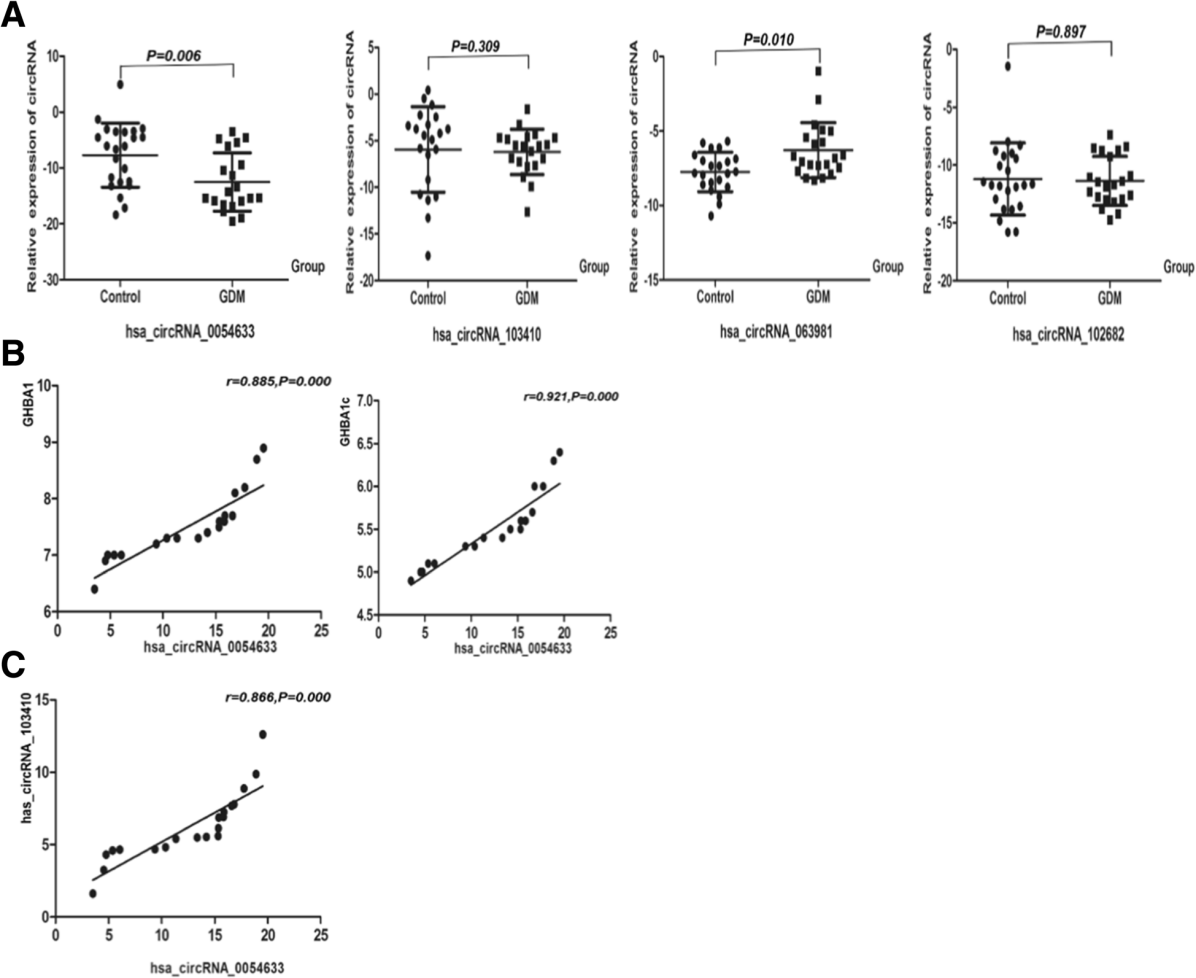
**a** Levels of circRNAs in fetal cord blood serum of healthy individuals (control; *n* = 20) and maternal gestational diabetes (GDM; *n* = 20). **b** Correlation between hsa_circRNA_0054633 level in fetal cord blood serum and maternal GHBA1 and GHBA1c in the group of mothers with gestational diabetes. **c** Correlation between the levels of the circRNAs in fetal cord blood serum of the GDM women

As shown in Fig. 3, the level of hsa_circRNA_0054633 in cord blood was significantly higher in the controls than in the GDM group (*P* < 0.05), while the level of hsa_circRNA_063981 was lower than that in the GDM group (*P* < 0.05). Next, we analyzed the correlation between the levels of these circRNAs and between the circRNA level and laboratory biochemical indicators. The level of hsa_circRNA_0054633 was highly correlated with maternal GHBA1 and GHBA1c, and the correlation coefficients were 0.885 and 0.921, respectively (*P* < 0.001, Additional file 1: Table S3). The level of hsa_circRNA_0054633 was correlated with that of hsa_circRNA_103410 (*P* = 0.000, *r* = 0.866) in the neonatal cord blood of the GDM group. However, there were no significant correlations between other circRNAs and clinical biochemical indicators.

### Hsa_circRNA_0054633 was highly expressed in placental tissues of the GDM study group and related to the development of GDM

**Fig. 4:**
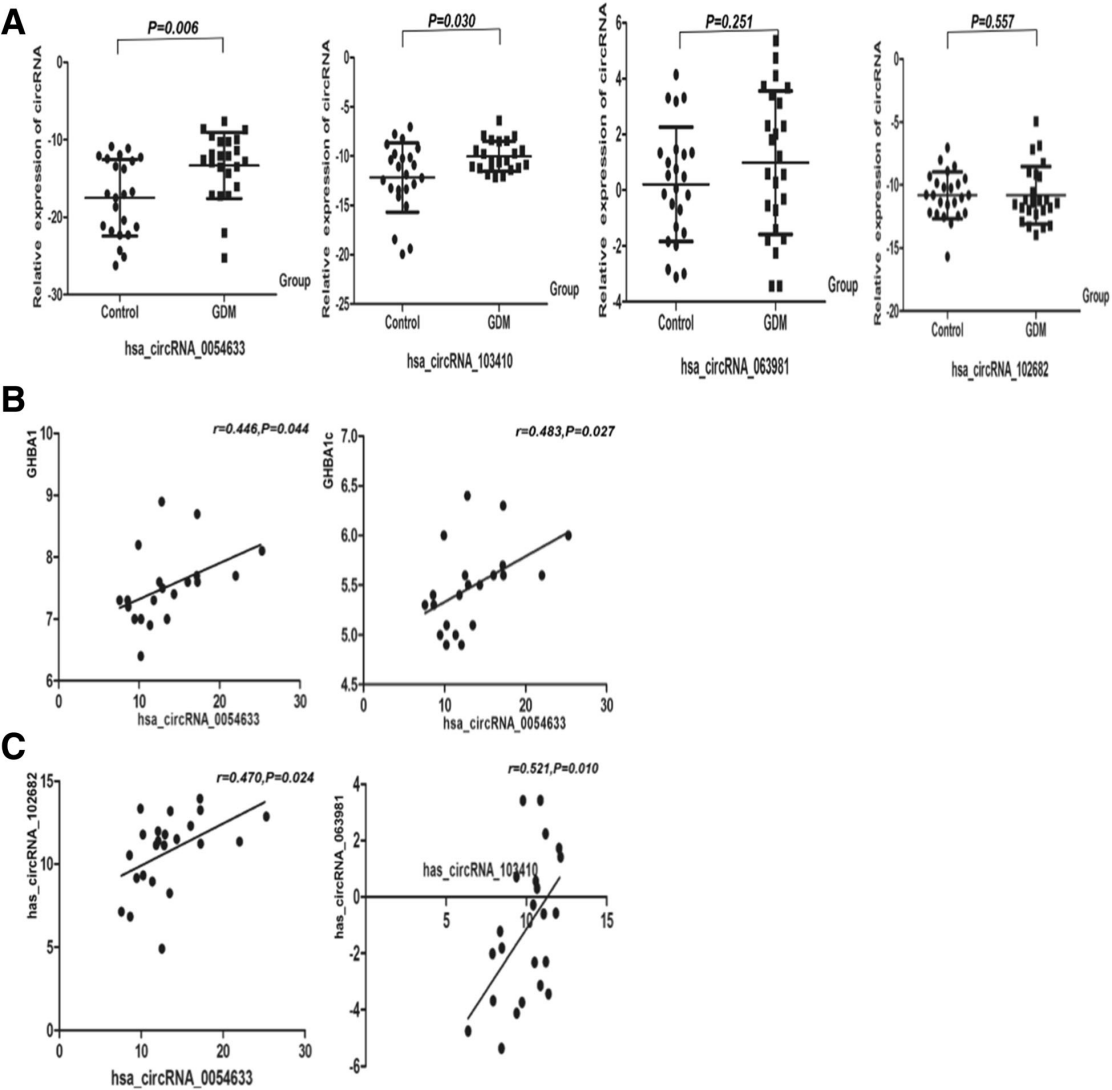
**a** Levels of circRNAs in placenta tissues of healthy individuals (controls; *n* = 20) and gestational diabetes (GDM; *n* = 20). **b** Correlation between the hsa_circRNA_0054633 level in the placenta and maternal GHBA1 and GHBA1c in the GDM group. **c** Correlation of between the levels of the circRNAs in placental tissues of the GDM women

The expression of hsa_circRNA_0054633 in the placental tissue of the GDM group was significantly higher than that of the healthy group. In addition, hsa_circRNA_103410 expression was higher in the GDM placental tissue than in the control placental tissue (*P* < 0.05, Fig. 4). Correlation analysis showed that the expression of hsa_circRNA_0054633 in the placenta of the case group was correlated with the levels of GHBA1 and GHBA1c in maternal blood (*P* < 0.05, *r* = 0.446 and *r* = 0.483, respectively). To determine if there is a correlation between circRNAs, we analyzed the correlation between the expression of different circRNAs and found that hsa_circRNA_0054633 and hsa_circRNA_102682 levels (*P* = 0.024, *r* = 0.470), as well as hsa_circRNA_103410 and hsa_circRNA_063981 levels, were correlated (*P* = 0.010, *r* = 0.521).

### ROC curve analysis of differentially expressed circRNAs

**Fig. 5:**
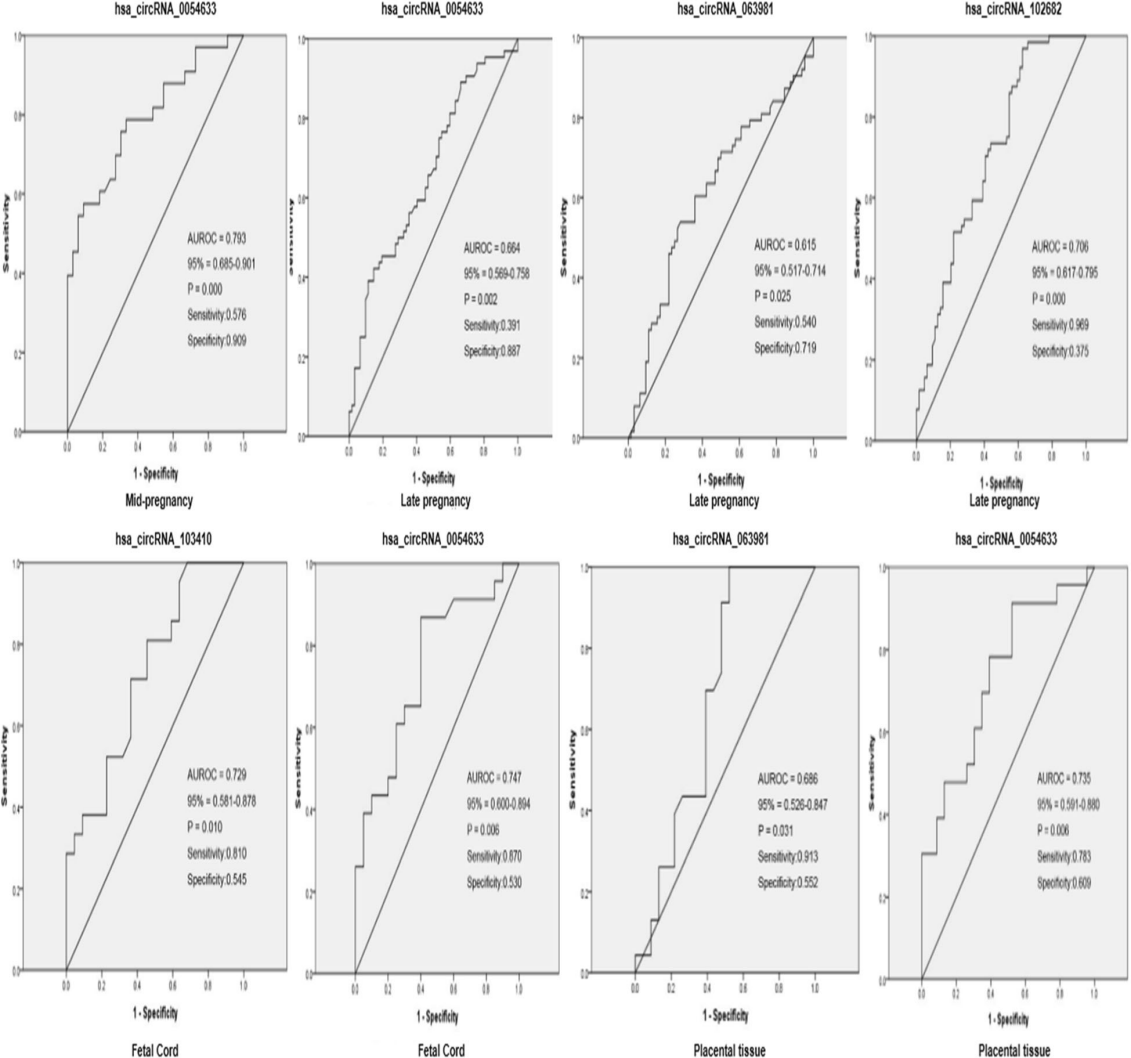
Diagnostic values of circRNAs in the second trimester blood, third trimester blood, fetal cord blood, or placental tissues as markers for gestational diabetes and possible adverse effects of the fetus. Abbreviation: AUROC, the area under the receiver operating characteristic curve; CI, confidence interval

The area under the ROC curve was used to determine the diagnostic value of circRNAs for GDM and neonatal complications (Fig. 5). The area under the curve (AUC) of hsa_circRNA_0054633 during the second trimester was 0.793 (0.685–0.901, *P* = 0.000); its best sensitivity and specificity were 57.6% and 90.9%, respectively. In the third trimester, the AUC of hsa_circRNA_0054633, hsa_circRNA_063981, and hsa_circRNA_102682 was 0.664 (0.569–0.758, *P* = 0.002), 0.615 (0.517–0.714, *P* = 0.025), and 0.706 (0.617–0.795, *P* = 0.000), respectively; the sensitivity and specificity were 39.1% and 88.7%, 54.0% and 71.9%, and 96.9% and 37.5%, respectively. In umbilical cord blood, the AUC of hsa_circRNA_103410 and hsa_circRNA_0054633 was 0.729 (0.581–0.878, *P* = 0.010) and 0.747 (0.600–0.894, *P* = 0.747), respectively. Furthermore, the AUC of hsa_circRNA_0054633 in the placental tissue was 0.735 (0.591–0.880, *P* = 0.006), with a sensitivity and specificity of 78.3% and 60.9%, respectively. The AUC of hsa_circRNA_063981 was 0.686 (0.526–0.847, *P* = 0.031); its sensitivity and specificity were 91.3% and 55.2%, respectively.

## Discussion

Gestational diabetes is very common and often has serious adverse consequences for the mothers and children. Similar to other reports, we also found that the GDM group had a higher weight and higher incidence of neonatal hypoglycemia than the healthy group [21, 22]. The BMI and total cholesterol level of GDM patients in the second and third trimesters were significantly higher than that of the controls, while HDL was lower in the cases than in the controls. During pregnancy, lipid levels gradually increase with gestational age [23, 24], and pregnancy is considered to be a condition of hyperlipidemia [25]. The United States National Cholesterol Education Program [26] has reported that patients with GDM have hypercholesterolemia, and earlier studies have suggested that non-coding RNAs are involved in regulating lipid homeostasis [27]. We analyzed the relationship between the tested circRNAs and lipid metabolism and found no association. Smolarczyk et al. [25] found that there was a positive correlation between estrogen levels in pregnant women and total lipid levels. However, the underlying cause of elevated blood cholesterol levels in GDM has not been fully elucidated.

In recent years, more and more researchers have proposed that epigenetic players such as non-coding RNAs are involved in the regulation of glucose metabolism in GDM [11, 28, 29]. CircRNAs are much more stable than linear RNAs in the cells. In some tissues, their expression levels are 10 times higher than linear RNAs [30]. Therefore, circRNAs are potentially better biomarkers than linear RNAs. We selected six circRNAs as candidate biomarkers and tested them in GDM patients. We found that hsa_circRNA_0054633 had the highest diagnostic value in GDM. Hsa_circRNA_0054633 was highly expressed in the serum in the second and third trimesters and also in the placenta and was positively correlated with postprandial blood glucose, GHBA1, and GHABA1c (*P* < 0.05). Zhao et al. showed that hsa_circRNA_0054633 is involved in biological processes such as cell cycle progression and also closely related to molecular catabolism [31]. The proliferation of pancreatic β cells is regulated by cell cycle progression, and a decrease in β cell proliferation is the main cause of diabetes where insulin secretion is insufficient [32]. Therefore, our results suggest that hsa_circRNA_0054633 may be involved in the regulation of pancreatic β cell proliferation and glucose metabolism during pregnancy. In addition, epigenetic changes in the placenta reflect the maternal diet and GDM metabolism [19]. We found that hsa_circRNA_0054633 is highly expressed in the placental tissues of the GDM group. Our finding suggests that hsa_circRNA_0054633 further affects the epigenetic changes in the placenta during its involvement in the regulation of glucose metabolism in maternal serum. However, placental factors can often lead to poor pregnancy outcomes which is associated with early placental development defects, and placenta inflammation, leading to increased risk of miscarriage, stillbirth, and fetal growth restriction [33]. Furthermore, we found that GDM cord blood has low levels of hsa_circRNA_0054633, suggesting that GDM can cause epigenetic changes in the cord blood, which might affect neonates. The specific mechanism needs to be further studied. By analyzing the area under the ROC curve in serum samples and placentas, we find that the diagnostic value of hsa_circRNA_0054633 for GDM was the highest among all circRNAs tested. Compared with known biomarkers, such as plasma protein profiling in the second trimester and hair metabolomics, hsa_circRNA_0054633 is more stable and highly sensitive and may predict neonatal complications. Hsa_circRNA_0054633 may participate in the development of GDM by affecting cell metabolism and cell cycle in early pregnancy and may further affect neonates through epigenetic changes in the placenta and cord blood. However, the specific role of this circRNA in GDM requires further investigation. There was a strong correlation between hsa_circRNA_0054633 and hsa_circRNA_103410 in the third-trimester maternal blood and in the umbilical cord blood, while hsa_circRNA_103410 only showed significant differences in the placenta tissue between GDM patients and healthy subjects. The hsa_circRNA_063981 level was low in the serum of GDM patients during the third trimester but high in the cord blood, and its expression in the placenta was correlated with hsa_circRNA_103410 expression (*P* < 0.05). Gu et al. [34]. proposed in a study that has_circRNA_063981 and hsa_circRNA_103410 participate in and regulate diabetic retinopathy. Our results suggest that these three circRNAs interact with each other and may cause adverse outcomes by modulating glycometabolism. The specific mechanism needs to be further studied.

In conclusion, hsa_circRNA_0054633 is highly expressed in GDM at different stages of pregnancy and is closely related to glycosylation markers. This study determined that hsa_circRNA_0054633 is a potentially highly sensitive serum biomarker for GDM. This finding opens up possibilities for the diagnosis of GDM and treatment by regulating the expression of hsa_circRNA_0054633.

## Materials and methods

### Subjects

A retrospective case-control study was conducted at the Ningbo University Medical Center Li Huili Eastern Hospital, China. The time the patients were diagnosed with GDM was from July 10, 2017, to February 15, 2018. Specimens included maternal serum samples, fetal cord blood, and placental tissue. Maternal serum samples were from the second trimester (24–28 weeks) and third trimester (36–41 weeks). Fetal cord blood and placental tissues were extracted immediately after delivery. In this study, we excluded women who had other pregnancy-associated diseases, chronic hypertension, multiple pregnancies, gynecological diseases, liver or kidney disease, cancer, pre-pregnancy type 1 or type 2 diabetes, or obesity (BMI ≥ 30). Those who were smoking were also excluded. This study was approved by the Ethics Committee of the Medical College of Ningbo University. During the collection of specimens for this study, all participants provided informed consent to use their blood and tissue samples in research. Pregnant women were screened for GDM at 24 to 28 weeks of gestation. If the fasting plasma glucose (FPG) level was 5.1 mmol/L or higher, GDM was diagnosed. GDM was ruled out if the FPG level was 4.4 mmol/L or less. Women whose FPG level was greater than 4.4 mmol/L but less than 5.1 mmol/L underwent a 75-g oral glucose tolerance test (OGTT). In this case, GDM was diagnosed when 1-h OGTT ≥ 10.0 mmol/L or 2-h OGTT ≥ 8.5 mmol/L. In this study, none of the subjects in the GDM group were given drugs or insulin injections to control blood glucose levels.

Specimens were collected from four cohorts: the second trimester (24–28 weeks) cohort included 40 GDM females (cases) and 40 healthy pregnant women (controls); the third trimester (36–41 weeks) cohort included 65 GDM patients and 65 healthy pregnant women; umbilical cord blood was from 20 GDM cases and 20 women without GDM; and placental tissues were from another 20 GDM cases and 20 women without GDM. Cases and controls were matched by age and BMI in all cohorts. Plasma hemoglobin levels were measured using a commercially available photometric method (SYSMEX, Hyôgo-ken, Japan). Blood biochemistry markers, including alanine aminotransferase (ALT), low-density lipoprotein (LDL), triglycerides, total cholesterol, and high-density lipoprotein (HDL), were assessed using a chemical analyzer (Siemens, Shanghai, China).

### Specimen collection and preservation

Serum samples of pregnant women were collected from the Li Huili Eastern Hospital of Ningbo University Medical Center from July 10, 2017, to February 15, 2018. In the second trimester (24–28 weeks) and third trimester (36–41 weeks), peripheral venous blood (3 mL) was collected and then centrifuged (3000 rpm for 10 min at room temperature). The serum was then immediately collected and stored at − 80 °C until use. Fetal umbilical cord blood (3 ml) was collected immediately after the child was born, and then, the serum was collected by centrifugation and stored at − 80 °C until use. After the placenta was delivered, placental leaflets about 1 × 1 × 1 cm below the umbilical cord were quickly excised into sterile RNase-free 2.0-ml centrifuge tubes and flash frozen in liquid nitrogen. These specimens were then stored at − 80 °C until use.

### Total RNA extraction and cDNA synthesis

Total RNA was isolated from the serum and placental tissue using TRIzol LS Reagent (Invitrogen, Karlsruhe, Germany) and TRIzol Reagent (Invitrogen), respectively, according to the manufacturer’s instructions. The purity of the extracted RNA was measured by a UV spectrophotometer using the following criteria: the 260/280 nm absorbance ratio of a qualified sample should be between 1.8 and 2.1. Among the extracted RNA samples, the number of sample failures in the second trimester and third trimester was kicked out by six and nine, respectively. RNA was reverse-transcribed into cDNA using HiFi-MMLV cDNA first strand synthesis kit (CWBIO) under the following conditions: 42 °C for 50 min, 85 °C for 5 min, and immediately stored at − 80 °C until use.

### Quantitative reverse transcription-polymerase chain reaction (qRT-PCR)

For quantification, real-time PCR analysis was performed using the LightCycler 480 SYBR Green I Master kit on a LightCycler 480 II (Roche). qRT-PCR was performed under the following conditions: 95 °C for a 5 min initial denaturation step, followed by 45 cycles of 94 °C for 10 s, primer pair-specific annealing temperature for 20 s, and 72 °C for 30 s. Primers are listed in Additional file 1: Table S1. The annealing temperature for hsa_circRNA_0054633, hsa_circRNA_0018508, and hsa_circRNA_103410 was 59 °C; for hsa_circRNA_063981 was 55 °C; for hsa_circRNA_102682 was 59 °C; and for β-actin and hsa_circRNA_104820 was 56 °C [31, 34, 35]. The relative fold changes were calculated by the comparative threshold cycle method, and β-actin was used as the internal normalization control. The experiment was performed independently three times.

### Statistical analyses

Statistical analysis of the data was performed using GraphPad Prism 5.0 for Windows and SPSS software version 18.0. Variables with different distributions were expressed as means ± standard deviations, medians (quartiles), or percentages. All data were first tested using the Kolmogorov-Smirnov test to see if the variables were normally distributed. When the variables satisfied the normal distribution, homogeneity tests for variance were performed. Independent samples *t* tests were used when normal distribution and homogeneity of variance were both satisfied. If one of the conditions was not met, the Mann-Whitney *U* test was used. When comparing the frequency of categorical variables, we used the Pearson’s *χ*^2^ test. *P* < 0.05 was considered statistically significant. The correlation between two variables was analyzed using the Pearson correlation test. The expression level of a gene was represented by the following formula: Δ*C*_T_ = *C*_T_ (target gene) − Δ*C*_T_ (internal reference gene), ΔΔ*C*_T_ = Δ*C*_T_ (case group gene of interest) − Δ*C*_T_ (control group gene of interest). When ΔΔ*C*_T_ < 0, the expression of the target gene was upregulated in the case group, and the expression was downregulated when ΔΔ*C*_T_ > 0. To determine the diagnostic value of circRNA for gestational diabetes in the four study cohorts, we established a ROC curve for each study cohort. The AUC was calculated for each circRNA.

## Additional file

Additional file 1: **Figure S1.** Hsa_circRNA_0018508 is not expressed during the third trimester. T1 and T2 represent the second trimester and the third trimester, respectively. (A) The level of hsa_circRNA_0018508 in the peripheral blood of pregnant women during the third trimester shown by agarose gel electrophoresis. (B) The amplification curves of fluorescent quantitative PCR. The red boxes in the figure represent the selected samples. **Table S1.** Primers for analysis of long non-coding RNAs by quantitative reverse transcription-polymerase chain reaction. **Table S2.** Differentially expressed circulating candidate circular RNA^a^. **Table S3.** Correlation between circRNA and Laboratory Parameters in Different Periods. (DOCX 293 kb)

## Abbreviations

ALT: Glutamic pyruvic transaminase
APOA1: Apolipoprotein A1
APOB: Apolipoprotein B
APOE: Apolipoprotein E
AST: Glutamic oxaloacetic transaminase
BMI: Body mass index
CircRNA: Circular RNA
HDL-C: High-density lipoprotein cholesterol
LDL-C: Low-density lipoprotein cholesterol
OGTT: Oral glucose tolerance test
TBA: Total bile acid
