# Supplementary material for: Hsa_circRNA_0054633 is highly expressed in gestational diabetes mellitus and closely related to glycosylation index
Source: Clin Epigenetics. 2019 Feb 8;11:22. doi: 10.1186/s13148-019-0610-8 (PMC6368772; doi:10.1186/s13148-019-0610-8)
Supplement: Supplementary file 1 — Figure S1. Hsa_circRNA_0018508 is not expressed during the third trimester. T1 and T2 represent the second trimester and the third trimester, respectively. (A) The level of hsa_circRNA_0018508 in the peripheral blood of pregnant women during the third trimester shown by agarose gel electrophoresis. (B) The amplification curves of fluorescent quantitative PCR. The red boxes in the figure represent the selected samples. Table S1. Primers for analysis of long non-coding RNAs by quantitative reverse transcription-polymerase chain reaction. Table S2. Differentially expressed circulating candidate circular RNAa. Table S3. Correlation between circRNA and Laboratory Parameters in Different Periods. (DOCX 293 kb) [file 13148_2019_610_MOESM1_ESM.docx]

**Table S1** Primers for analysis of long non-coding RNAs by quantitative reverse transcription polymerase chain reaction.

| **Primer and orientation** | **Sequence** |
| --- | --- |
| β-actin ^a^ |  |
| Forward | GCCGATCCACACGGAGTACTT |
| Reverse | TTGCCGACAGGATGCAGAA |
| hsa_circRNA_0054633 |  |
| Forward | TTGCTTTCTACACTTTCAGGTGAC |
| Reverse | GCTTTTTGTCTGTAGTCAACCACCA |
| hsa_circRNA_406918 |  |
| Forward | GTCTGTTCCCACCCACTTCA |
| Reverse | GTCTAGTGCTCTCAAACTGCGG |
| hsa_circRNA_103410 |  |
| Forward | GGCGCTGCTTCATCACAT |
| Reverse | TCGGCAAGTCCTCAAAACC |
| has_circRNA_102682 |  |
| Forward | GTTTCTCTGAGTCCTGCCCT |
| Reverse | GGATTGCTGCAGGTTCGAAT |
| hsa_circRNA_0018508 |  |
| Forward | TCTTTGCCACATATTGGGTGACT |
| Reverse | ACACCAGGTACCGGTTATCCA |
| hsa_circRNA_063981 |  |
| Forward | CCACGACATCCACCTCCTC |
| Reverse | ACTCCAGCTCCGGGTCCA |
| Abbreviation: circRNA, circular RNA. | |
| ^a^ Used as the internal normalization control. | |

**Table S2**  Differentially expressed circulating candidate circular RNA.^a^

| **Mid-pregnancy blood samples** | **Healthy control group (n=40)** | **Gestational diabetes mellitus group(n=40)** | ***P* value ^b^** |
| --- | --- | --- | --- |
| hsa_circRNA_0054633 | -5.77±3.00 | -2.05±3.27 | 0.000 * |
| hsa_circRNA_0018508 | -4.58±2.38 | -3.97±1.94 | 0.279 |
| hsa_circRNA_103410 | -3.13±2.60 | -2.56±1.91 | 0.302 |
| hsa_circRNA_063981 | -2.21±2.00 | -2.28±1.97 | 0.768 |
| has_circRNA_102682 | -7.12±1.59 | -6.86±1.80 | 0.526 |
| hsa_circRNA_406918 | -3.91±2.35 | -3.59±1.71 | 0.520 |
| **Late pregnancy blood samples** | **Healthy control group (n=65)** | **Gestational diabetes mellitus group(n=65)** | ***P* value ^b^** |
| hsa_circRNA_0054633 | 2.35±1.36 | 3.05±2.00 | 0.002 * |
| hsa_circRNA_103410 | -7.85±3.68 | -7.43±3.22 | 0.553 |
| hsa_circRNA_063981 | -1.13±1.87 | -1.69±1.77 | 0.025 * |
| has_circRNA_102682 | -6.38±2.23 | -8.59±3.10 | 0.000 * |
| hsa_circRNA_406918 | -6.71±4.20 | -7.61±4.86 | 0.281 |
| **Fetal cord blood samples** | **Healthy control group (n=20)** | **Gestational diabetes mellitus group(n=20)** | ***P* value ^b^** |
| hsa_circRNA_0054633 | -7.71±5.74 | -12.51±5.21 | 0.006 * |
| hsa_circRNA_103410 | -5.94±4.60 | -6.19±2.44 | 0.309 |
| hsa_circRNA_063981 | -7.75±1.33 | -6.28±1.85 | 0.010 * |
| has_circRNA_102682 | -11.20±3.12 | -11.36±2.12 | 0.897 |
| hsa_circRNA_406918 | -8.36±2.33 | -7.74±3.25 | 0.192 |
| **Placenta samples** | **Healthy control group (n=20)** | **Gestational diabetes mellitus group(n=20)** | ***P* value ^b^** |
| hsa_circRNA_0054633 | -17.50±4.93 | -13.32±4.29 | 0.006 * |
| hsa_circRNA_103410 | -12.17±3.51 | -10.02±1.52 | 0.030* |
| hsa_circRNA_063981 | -0.21±2.05 | -0.99±2.56 | 0.251 |
| has_circRNA_102682 | -10.81±1.85 | -10.80±2.28 | 0.557 |
| hsa_circRNA_406918 | -5.79±0.53 | -5.92±1.30 | 0.138 |
| ^a^ Values are given as mean ± SD or number (percentage), unless otherwise indicated.  ^b^ *p-value from t-test or nonparametric test comparing means or proportions among GDM cases and controls in the current study.P<0.05 was considered statistically significant. | | | |

**Table S3** Correlation between circRNA and Laboratory Parameters in Different Periods.

| **Mid-pregnancy** | **Variable** | **Correlation coefficient** | ***P* value** |
| --- | --- | --- | --- |
| hsa_circRNA_0054633 | 2-h glucose ,mmol/L | 0.532 | 0.000* |
|  | GHBA1,% | 0.318 | 0.043* |
|  | GHBA1c,% | 0.331 | 0.040* |
| **Cord blood** | | | |
| hsa_circRNA_0054633 | GHBA1,% | 0.885 | 0.000* |
|  | GHBA1c,% | 0.921 | 0.000* |
| **Placental tissue** | | | |
| hsa_circRNA_0054633 | GHBA1,% | 0.446 | 0.044* |
|  | GHBA1c,% | 0.483 | 0.027* |
| All variables were tested using linear correlation analysis;P<0.05 was considered statistically significant. | | | |

**Figure S1**


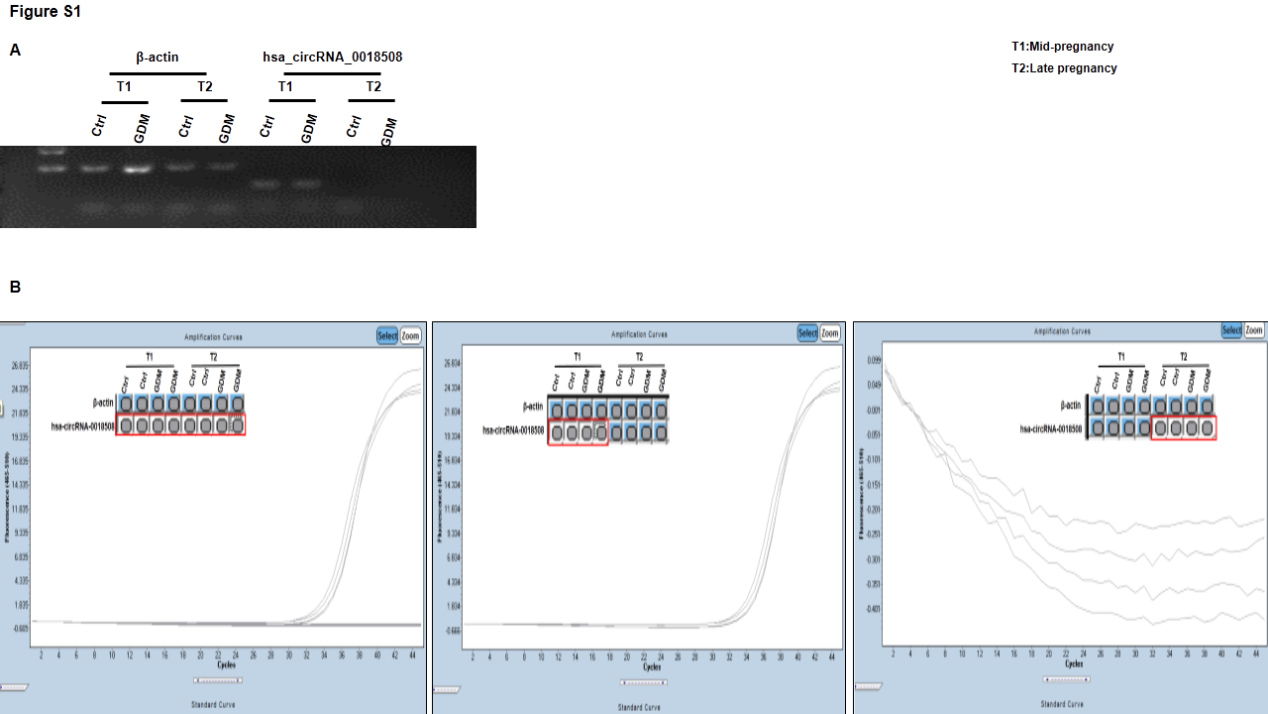


**Figure legends**

**Figure S1** Verify that hsa_circRNA_0018508 is not expressed during late pregnancy. In the figure, T1 and T2 represent the second trimester and the third trimester, respectively. (A) The expression of hsa_circRNA_0018508 in the peripheral blood of pregnant women during the third trimester was verified by agarose gel electrophoresis. (B) The amplification curve was observed by fluorescence quantitative PCR to show that hsa_circRNA_0018508 was not expressed in late pregnancy. The red box in the figure represents the selected sample.
